# Putative role of HLA polymorphism among a Brazilian HTLV-1-associated myelopathy/tropical spastic paraparesis (HAM/TSP) population

**DOI:** 10.1038/s41598-023-34757-w

**Published:** 2023-05-11

**Authors:** Doris Schor, Luís Cristóvão Porto, Eric Henrique Roma, Julio Castro-Alves, Anna Paula Villela, Abelardo Q. C. Araújo, Maria Glória Bonecini-Almeida

**Affiliations:** 1 Laboratório de Imunologia e Imunogenética em Doenças Infecciosas, Instituto Nacional de Infectologia Evandro Chagas-INI/FIOCRUZ, Avenida Brasil, 4365, Manguinhos, Rio de Janeiro, RJ 21040-900 Brazil; 2 Laboratório de Histocompatibilidade e Criopreservação, Universidade do Estado do Rio de Janeiro (UERJ), Rio de Janeiro, RJ 20950-000 Brazil; 3 Plataforma de Pesquisa Clínica, Instituto Nacional de Infectologia Evandro Chagas-INI/FIOCRUZ, Rio de Janeiro, RJ 21040-900 Brazil; 4 Laboratório de Pesquisa Clínica em Neuroinfecções, Instituto Nacional de Infectologia Evandro Chagas-INI/FIOCRUZ, Rio de Janeiro, RJ 21040-900 Brazil

**Keywords:** Immunogenetics, HTLV

## Abstract

Around ten million people are infected with HTLV-1 worldwide, and 1–4% develop HTLV-1-associated myelopathy/tropical spastic paraparesis (HAM/TSP), characterized by an important degeneration of the spinal cord, which can lead to death. Distinct HLA alleles have been associated with either HAM/TSP susceptibility or protection. However, these HLA alleles set may change according to the population studied. Brazil is the second country in the number of HTLV-1-infected people and there are few reports addressing the HLA influence on HTLV-1 infection as well as on disease outcome. The objective of this study was to evaluate the influence of HLA alleles as a risk factor for HAM/TSP and the proviral load (PVL) levels, clinical progression, and death outcomes in an admixed Brazilian population. The HLA-A, -B, -C, and -DRB1 were genotyped in 375 unrelated HTLV-1-infected individuals divided into asymptomatic carriers (AC) (n = 165) and HAM/TSP (n = 210) in a longitudinal cohort from 8 to 22 years of follow-up. Because locus B deviated from Hardy–Weinberg Equilibrium for the study groups, the results represented for HLA-B alleles were inconclusive. The alleles HLA-A*68 and -C*07 were related to HAM/TSP risk in multivariate analysis. The alleles HLA-A*33, and -A*36 were associated with protection against disease progression in HAM/TSP patients, while -C*12, -C*14, and -DRB1*08 were associated with increased risk of death. In the AC group, the presence of, -C*06 and -DRB1*15 alleles influenced an increased PVL, in an adjusted linear regression model, while -A*30, -A*34, -C*06, -C*17 and -DRB1*09 alleles were associated with increased PVL in HAM/TSP group compared to HAM/TSP individuals not carrying these alleles. All these alleles were also related to increased PVL associated with clinical progression outcome. Increased PVL associated with the death outcome was linked to the presence of HLA-A*30. PVL has been associated with HLA, and several alleles were related in AC and HAM/TSP patients with or without interacting with clinical progression outcomes. Understanding the prognostic value of HLA in HAM/TSP pathogenesis can provide important biomarkers tools to improve clinical management and contribute to the discovery of new therapeutic interventions.

## Introduction

The human T cell lymphotropic virus (HTLV-1) is a retrovirus associated with two primary diseases: adult T cell leukemia/lymphoma (ATLL)^1^ and the HTLV-1-associated myelopathy/tropical spastic paraparesis (HAM/TSP)^2,3^. Approximately 5 to 10 million people are infected with HTLV-1 worldwide^4^. Japan and Brazil are countries with the highest estimated total numbers of HTLV-1-infected people^5^. The HTLV-1 prevalence in Brazil ranges from 146.3 to 390.2/100,000 inhabitants^4^. Although most infected individuals remain asymptomatic, around 0.25–3.8% develop HAM/TSP over time^6^. This neurological disease is characterized as a slowly progressing spinal cord disease. The leg muscles gradually become weak. The limbs are stiff, the movements are slow, and walking becomes more difficult. Muscle spasms in the legs are common. Other HTLV-1-associated diseases, such as neurogenic bladder^7^ and ocular manifestations^8^, can also be present among HTLV-1-positive individuals. These clinical outcomes are associated with increased HTLV-1 proviral loads (PVL)^9^; however, it is still unclear what determines the high PVL in these patients. The feature that only a small percentage of HTLV-1 carriers will develop some symptoms has not yet been fully elucidated.

The neurological injury in HAM/TSP may be a consequence of an inflammatory reaction triggered by the recognition of infected cells by cytotoxic T lymphocytes, followed by the release of cytokines and damage to the central nervous system. Factors related to the HTLV-1/host interaction may be involved in the risk of developing HAM/TSP^9^.

In the search for a biomarker that can identify who is more prone to develop the disease, depending on the population's geographic distribution, some studies have shown that HLA alleles can influence the course of the infection.

The HLA alleles A*31, B*07, B*54, C*07, DRB1*01, and DQB1*05 were described to be related to a higher risk of developing HAM/TSP in Japan^10–14^. DRB1*01, in the absence of A*02 and C*08, was related to a higher risk of developing HAM/TSP in the Iranian population^15,16^. In addition, B*07 and DRB1*01 were related to an increased risk of developing HAM/TSP in Spain, where the majority of the individuals studied were from Latin America^17^. In South America, the allele B*35 was associated with a higher risk of developing HAM/TSP in Argentina^18^. On the other hand, the HLA-A*02, -C*08, -B*40, -DRB1*15, and -DQB1*06 were related to protection in Japan^11–13^. However, in Iran, Caribbean, Peruvian, and Spain populations, the same HLA alleles were unrelated to HAM/TSP susceptibility or protection^15–17,19–22^. Only two studies were performed in Brazil, involving a very small sample size of patients with HAM/TSP (n = 9) or selecting alleles previously associated with protection and disease risk in other geographic populations^23,24^. Unfortunately, these studies did not assess the complete HLA alleles or compare the clinical outcome. In addition, the Brazilian population is highly admixed and a probable association of ethnicity should be considered. Our objective was to describe the genetic polymorphism in the HLA-A, -B, -C, and -DRB1 loci in a subset of the Brazilian population infected with HTLV-1 in a longitudinal follow-up cohort at a Reference Center for Infectious Diseases in Rio de Janeiro, Brazil.

## Results

### Patients and clinical data

Table 1 contains demographic and clinical data. Three hundred and seventy-five individuals infected with HTLV-1 were randomly selected from the HTLV-1 cohort, corresponding to 41.7% of all HTLV-1 infected individuals followed as outpatients at the INI/Fiocruz. The enrolled participants lived predominantly in the metropolitan area of Rio de Janeiro City. They were divided into HAM/TSP (n = 210) and AC (n = 165) subgroups. The median age of the HAM/TSP group was 61 (59.33–62.55, 95% CI) years, similar to the AC group (61 (54.92–59.82 years, *p* = 0.14). The median clinical follow-up time was 13 (12.24–13.96, 95% CI), and 17 (14.29–16.57) years for HAM/TSP and AC, respectively. The distribution of gender and ethnicity (a self-identified skin color, described as white or non-white (black and mixed)) was similar in both groups. Also, the HAM/TSP group presented a higher frequency of other clinical manifestations such as the neurogenic bladder (79.5%) and ophthalmic alterations (10.5% of the patients) compared to the AC group, *p* < 0.01. PVL was higher in the HAM/TSP group compared to the AC group (p < 0.01).

**Table 1:** Demographic and clinical data in HAM/TSP and AC Brazilian patients.

	HAM/TSP (n = 210)	AC (n = 165)	OR (95% CI)/mean ± SD	P-value
Sex (n, %)				
Female	132 (62.9)	90 (54.5)	–	–
Male	78 (37.1)	75 (45.5)	0.71 (0.47–1.07)	0.1
Age (years)				
Median (95% CI)	61 (59.33–62.55)	61 (54.92–59.82)	–	0.14
Ethnicity (n, %)^$^				
White	103 (49.0)	77 (46.7)	–	–
Non-white	106 (50.5)	88 (53.3)	0.9 (0.6–1.36)	0.62
N/D	1 (0.5)	0 (0.0)		
Clinical follow-up (years)				
Median (95% CI)	13 (12.24–13.96)	17 (14.29–16.57)	–	** < 0.01**
Clinical progression (n, %)^$^				
No	34 (16.2)	152 (92.2)	–	–
Yes	148 (70.5)	07 (4.2)	60.15 (29.38–123.15)	** < 0.01**
N/D	28 (13.3)	6 (3,6)		
IPEC-CDS motor subscore				
Median (95% CI)	18.65 (18.28–21.09)	0.0 (0.48–1.34)	–	** < 0.01**
Neurogenic bladder (n, %)^$^				
No	41 (19.5)	154 (93.3)	–	–
Yes	167 (79.5)	11 (6.7)	57.02 (28.3–114.9)	** < 0.01**
N/D	2 (1.0)	–		
Ophthalmic alterations (n, %)				
No	188 (89.5)	160 (97)	–	–
Yes	22 (10.5)	5 (3)	8.98 (3.48–23.17)	** < 0.01**
Proviral load (proviral copies/cell)				
Median (95% CI)	6.49 (7.40–9.31)	1.91 (3.16–5.18)	–	** < 0.01**
Death outcome (n, %)^$^				
No	164 (78.1)	160 (97.0)	–	–
Yes	24(11.4)	0 (0)	47.81 (2.88–793.4)	** < 0.0001**
N/D	22 (10.5)	5 (3.0)	–	

*AC* asymptomatic carrier, *HAM/TSP* human T cell lymphotropic virus type 1–associated myelopathy/tropical spastic paraparesis, *N/D* not determined, *OR* odds ratio and 95% confidence interval.

Significant values are in bold.

^$^Missing data (N/D) were not considered for OR calculation and statistical analysis.

During the clinical follow-up, 24 (11.4%) patients died from secondary causes related to HTLV-1 infection, such as recurrent urinary infections, bedsores, and sepsis. The most common causes of death among HAM/TSP in our cohort were urinary sepsis and deep venous thrombosis associated with pulmonary thromboembolism. All of these individuals were HAM/TSP patients. The clinical progression was investigated during the follow-up, and 148 (70.5%) HAM/TSP and only seven (4.2%) AC patients, showed a deteriorating outcome (*p* < 0.01). The clinical follow-up in the AC progressors (v.g., from AC to HAM/TSP) subgroup ranged from three to 26 years (median 22 years, 9.98–25.73, 95% CI).

**Figure 1:**
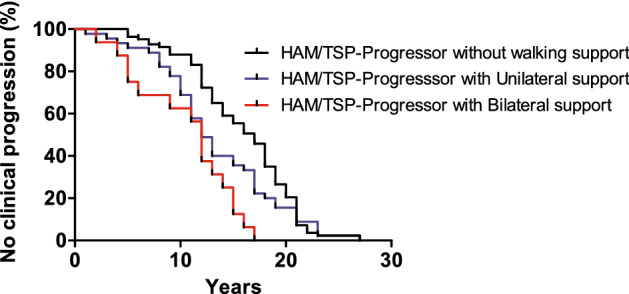
Longitudinal clinical progression in HAM/TSP patients related to walking condition ability. A log-Rank Mantel-Cox test evaluated the statistical difference between HAM/TSP Progressors subgroups. The Y-axis represents the percentage of patients without disease progression through the time (in years). The difference between the HAM/TSP-Progresssor with Unilateral support versus HAM/TSP-Progresssor with Bilateral support was statistically significant (*p* = 0.0462). The comparison of clinical progression between the HAM/TSP-Progressor without walking support and the HAM/TSP-Progresssor with Bilateral support group was also statistically significant (p < 0.0001). No difference was observed when comparing HAM/TSP-Progressor without walking support versus HAM/TSP-Progresssor with Unilateral support patients.

Figure 1 shows the clinical progression throughout 1–27 years (median 14 years, 13.53–15.34, 95% CI) of follow-up in the HAM/TSP progressors subgroup. The clinical progression occurred more rapidly according to the severity of the patient’s condition. Patients with the worse disability, such as those with bilateral walking support (HAM/TSP Bilateral-Progressors), progressed more quickly than those with unilateral walking support (HAM/TSP Unilateral-Progressors) or those able to walk without support (HAM/TSP walking). A Log-Rank Mantel-Cox test showed a statistically significant difference between subgroups (*p* < 0.0001). The difference in clinical evolution between those individuals with HAM/TSP with unilateral support and bilateral support was significant (p = 0.0462); however, there was no difference in clinical development between individuals who needed unilateral support and those with an independent gait.

### HLA genotyping

The frequencies of the HLA -A, -B, -C, and -DRB1 alleles in the study population were compatible with those found at the Brazilian Bone Marrow Volunteer Donor Registry (REDOME)^25^, updated to January 2023, except for the HLA-A*68 allele. From 5,585,608 HLA-typed Brazilians HLA-A*68 represented 6.20% and in the Rio de Janeiro State 6.41% among 254,519 donors, in contrast with 10.37% observed in our study population. Supplemental Table S1 described all allelic frequencies found in our study population.

The B locus deviated from the Hardy–Weinberg Equilibrium (HWE) for both HAM/TSP and AC groups (p < 0.0000 and p < 0.00088, respectively, Supplemental Tables S2 and S3), and for this reason, the results were inconclusive. The remaining loci are in HWE for both groups. After univariate and multivariate analysis, alleles HLA-A*68 and -C*07 showed a higher frequency in the HAM/TSP group than in the AC group, even after being adjusted by age, gender, ethnicity, and time of clinical follow-up (Table 2, OR 2.03 (1.19–3.46, 95% CI), *p* = 0.01, and OR 1.61 (1.04–2.48, 95% CI), *p* = 0.03, respectively), and were associated to HAM/TSP risk. As HLA-C*07 in the absence of -A*02 has been previously described as a risk factor for HAM/TSP^24^, we tested its association in the absence of the -A*02 allele. The contingency table analysis showed that the presence or absence of the -A*02 did not influence the -C*07 allele distribution in both groups.

**Table 2:** HLA allele’s frequency in HAM/TSP and AC Brazilian patients.

	HAM/TSP (n = 210)	AC (n = 165)	OR (95% CI)	P-value	OR-adjusted (95% CI)*	P-value*
HLA alelles n (%)						
-A*68	55 (13.3)	23 (7.3)	1.96 (1.17–3.26)	0.01	2.03 (1.19–3.46)	**0.01**
-C*07	63 (22.8)	47 (16.1)	1.54 (1.01–2.35)	0.04	1.61 (1.04–2.48)	**0.03**

*AC* asymptomatic carrier, *HAM/TSP* human T cell lymphotropic virus type 1-associated myelopathy/tropical spastic paraparesis, *OR* odds ratio 95% confidence interval.

Significant values are in bold.

*p values adjusted by age, sex, ethnicity and time of clinical follow-up.

Due to the small number of affected patients with neurogenic bladder and ophthalmological manifestations was not possible to correlate these data with HLA genotyping.

Predicted three loci haplotypes are represented in Supplemental Tables S4 and S5, for AC and HAM/TSP groups, respectively. In total, 209 and 196 HLA- A ~ C ~ B haplotypes were observed in AC and HAM/TSP patients, respectively. The most common (> 1%) haplotypes were restricted to a minimum of four (A01 ~ C07 ~ B08; A01 ~ C04 ~ B35; A02 ~ C05 ~ B44, A68 ~ C04 ~ B44; A02 ~ C14 ~ B51; A68 ~ C07 ~ B07, A68 ~ C07 ~ B07) copies in AC group, with maximum 11 copies for the A02 ~ C04 ~ B35) haplotype. The most common haplotype in the AC group was observed in only one copy in the HAM/TSP group. Otherwise, the most common haplotype in HAM/TSP (A01 ~ C07 ~ B08) was expressed in five copies in this group and four in the AC group. No differences were observed between both groups.

### HLA genotypes and clinical outcomes

Logistic regression was performed to analyze whether HLA-A, -C, and -DRB1 alleles were associated with different clinical progression outcomes in HAM/TSP patients. Only seven AC patients progressed to HAM/TSP during the clinical follow-up (17.86 ± 8.51 years), while 148 patients in HAM/TSP group showed clinical progression. Therefore, we only analyzed the association between HLA and disease progression in the HAM/TSP group (Table 3). The alleles -A*33 (OR 0.28 (0.09–0.91 95% CI), p = 0.03) and -A*36 (OR 0.12 (0.02–09 95% CI), p = 0.04) were associated with protection against disease progression in the adjusted model. However, these alleles have a low frequency in our population and these results should be tested in other populations. Supplemental Table S6 shows the frequency of all HLA-A, -B, -C, and -DRB1 loci in HAM/TSP patients related to the disease progression used in this study. Logistic regression was also performed to verify HLA alleles' association with death in the HAM/TSP group. The alleles -C*12 (OR 6.25 (1.71–22.8, CI 95%), *p* = 0.01), -C*14 (OR 8.85 (1.68–46.67, CI95%), *p* = 0.01) and -DRB1*08 (OR 9.1 (1.72–48.04, CI 95%), *p* = 0.01) were associated with death in HAM/TSP patients in the adjusted model (Table 4). Unfortunately, these alleles have a low frequency in our studied population and these results should be tested in other populations to prove their role in HAM/TSP death outcome. Supplemental Table S7 shows the frequency of all HLA-A, -B, -C and -DRB1 loci in HAM/TSP patients related to death used in this study.

**Table 3:** HLA allele´s frequency in HAM/TSP associated with disease progression.

	Progression					
	No (n = 34)	Yes (n = 148)	OR (95% CI)	P-value	OR-Adjusted (95% CI)*	P-value*
Alelles n (%)						
-A*33	6 (9)	7 (2.4)	0.25 (0.08–0.78)	**0.02**	0.28 (0.09–0.91)	**0.03**
-A*36	2 (3)	2 (0.7)	0.23 (0.03–1.64)	0.14	0.12 (0.02–0.9)	**0.04**

*HAM/TSP* human T cell lymphotropic virus type 1-associated myelopathy/tropical spastic paraparesis, *OR* odds ratio 95% confidence interval.

Significant values are in bold.

*p values adjusted by age, sex, ethnicity and time of clinical follow-up.

**Table 4:** HLA allele’s frequency in HAM/TSP associated with death.

	Death					
	No (n = 164)	Yes (n = 24)	OR (95% CI)	P-value	OR-Adjusted (95% CI)*	P-value*
Alelles n (%)						
-C*12	11 (4.5)	5 (15.6)	3.92 (1.27–12.14)	0.02	6.25 (1.71–22.8)	**0.01**
-C*14	4 (1.6)	4 (12.5)	8.57 (2.03–36.18)	< 0.01	8.85 (1.68–46.67)	**0.01**
-DRB1*08	9 (3.6)	3 (16.7)	5.36 (1.31–21.87)	0.02	9.1 (1.72–48.04)	**0.01**

*HAM/TSP* human T cell lymphotropic virus type 1-associated myelopathy/tropical spastic paraparesis, *OR* odds ratio 95% confidence interval.

Significant values are in bold.

*p values adjusted by age, sex, ethnicity and time of clinical follow-up.

### HLA genotypes, PVL, and its association with clinical progression outcome

Several HLA alleles have been associated with PVL in Japan^11,26^ but not in other populations. We analyzed whether HLA alleles in our population alter the PVL. We performed an independent case and control analysis because PVL was higher in the HAM/TSP group compared to the AC group (Table 1). Table 5 describes, through linear regression analysis, the role of the HLA alleles in the PVL in AC patients. Individuals in this group, who carry the alleles C*06 (β 3.904 (1.016; 6.792, 95% CI), *p* = 0.008) and -DRB1*15 (β 3.381 (0.741; 6.021, 95% CI), *p* = 0.012) had an increased PVL compared to those who do not carry those alleles (β, 95% CI as shown in Table 5), after adjustment. In the univariate analysis, carriers of the alleles -A*30 and -A*66 also presented higher PVL (Table 5). However, after adjusting the multivariate analysis, there was no difference between carriers and not-carriers in the PVL related to these alleles.

**Table 5:** Linear regression analysis of proviral load and HLA-A, -C and -DRB1 loci in AC Brazilian patients.

Alleles	Status			
	β (95% CI)	P-value	β (95% CI), adjusted*	P-value*
-A*30				
Alelle absent	–	–	–	–
Alelle present	3.128 (0.247;6.009)	0.033	2.56 (− 0.323;5.443)	0.081
-A*66				
Alelle absent	–	–	–	–
Alelle present	4.772 (0.167;9.378)	0.042	3.826 (− 0.774;8.426)	0.103
-C*06				
Alelle absent	–	–	–	–
Alelle present	3.912 (1.019;6.805)	0.008	3.904 (1.016;6.792)	**0.008**
-DRB1*15				
Alelle absent	–	–	–	–
Alelle present	3.407 (0.773;6.041)	0.011	3.381 (0.741;6.021)	**0.012**

*AC* asymptomatic carrier, *95% CI* confidence interval

Significant values are in bold.

*p values adjusted by age, sex, ethnicity and time of clinical follow-up.

In the HAM/TSP group, individuals who carry the A*30 (β 3.033 (0.252;5.8130, 95% CI), *p* = 0.033), -A*34 (β 9.172 (2.755;15.588, 95% CI), *p* = 0.005), -C*06 (β 4.016 (0.883;7.15, 95% CI), *p* = 0.012), -C*17 (β 7.048 (0.696;13.399, 95% CI), *p* = 0.03) and -DRB1*09 (β 6.874 (2.341;11.408, 95% CI), *p* = 0.003) alleles had an increased PVL compared to those who do not carry those alleles (β, 95% IC as showed in Table 6). Indeed, these alleles were also related to an increased PVL and a worse clinical progression (Table 6). On the other hand, only the allele -A*30 (β 24.634 (11.282; 37.985, 95% CI) was associated with a higher PVL and death (Table 6). Due to the low frequency of -DR*09 carries, it was not possible to calculate the β value in the multivariate model. None of the alleles -A*34, -C*06, -C*17, and -DRB1*09 were associated with both death outcome and the PVL levels in HAM/TSP patients.

**Table 6:** Linear regression analysis of proviral load and HLA- A, B, C and DR Loccus related to clinical progression outcomes in HAM/TSP Brazilian patients.

Alleles	PVL				PVL and clinical progression outcome				PVL and death			
	β(95% CI)	P-value	β (95% CI), adjusted*	P-value*	β(95% CI)	P-value	β (95% CI), adjusted*	P-value*	β(95%IC)	P-value	β (95% IC), ajusted	P-value
-A*30												
Alelle absent	–	–	–	–	–	–	–	–	–	–	–	–
Alelle present	3.563 (0.774;6.352)	0.012	3.033 (0.252;5.813)	0.033	5.828 (2.275;9.381)	0.001	5.369 (1.796;8.941)	0.003	25.208 (12.843;37.573)	0	24.634 (11.282;37.985)	0.001
-A*34												
Alelle absent	–	–	–	–	–	–	–	–	–	–	–	–
Alelle present	9.388 (2.884;15.892)	0.005	9.172 (2.755;15.588)	0.005	12.511 (2.943;22.08)	0.011	12.042 (2.507;21.578)	0.013	20.749 (0.881;40.617)	0.041	20.086 (− 1.138;41.31)	0.063
-C*06												
Alelle absent	–	–	–	–	–	–	–	–	–	–	–	–
Alelle present	4.284 (1.13;7.438)	0.008	4.016 (0.883;7.15)	0.012	5.866 (2.339;9.393)	0.001	5.519 (2.004;9.034)	0.002	15.746 (− 1.079;32.57)	0.065	10.361 (− 6.403;27.125)	0.213
-C*17												
Alelle absent	–	–	–	–	–	–	–	–	–	–	–	–
Alelle present	7.901 (1.499;14.302)	0.016	7.048 (0.696;13.399)	0.03	11.014 (3.757;18.272)	0.003	10.505 (3.277;17.733)	0.005	25.755 (3.167;48.344)	0.027	18.218 (− 4.302;40.737)	0.107
-DRB1*09												
Alelle absent	–	–	–	–	–	–	–	–	–	–	–	–
Alelle present	7.007 (2.413;11.601)	0.003	6.874 (2.341;11.408)	0.003	10.781 (5.498;16.065)	0.01	10.474 (5.246;15.703)	0.01	26.309 (6.586;46.032)	0.013	###	–

*HAM-TSP* human T cell lymphotropic virus-associated myelopathy/tropical spastic paraparesis, *95% CI* confidence interval.

*p values adjusted by age, sex, ethnicity and time of clinical follow-up, RC = xxx, ^###^there was no possible estimate the parameter in the multivariate model.

## Discussion

Brazil is a country with continental proportions and endemic to HTLV-1. Its population is composed of miscegenation among individuals of Indigenous, African, Asia, and European origin. Unfortunately, the Brazilian population is underrepresented in public genomic reference databases. Previous data comparing whole exome sequencing and SNP array from the Brazilian population and the 1000 Genomes datasets showed that there are significant differences in allele frequencies for both common and rare variants and this result indicates that none of the admixed American populations present in the 1000 Genomes dataset can be used as a surrogate for studies of the Brazilian population^27^. This is a limitation of our case–control study. Some studies have associated HLA genetic polymorphism with protection or susceptibility to HAM/TSP development. This study evaluated the influence of HLA class I and II gene polymorphism in HTLV-1 infected individuals in a longitudinal follow-up and its association with PVL levels, clinical progression, and death outcomes. AC and HAM/TSP patients were accompanied for two to a maximum of 27 years, and clinical progression was related to HAM/TSP patients. The lifetime risk of HAM/TSP is different among ethnic groups^26^. Some studies report that it can take up to 21 years from the onset of infection to using a wheelchair in HAM/TSP^28^. Our study could not specify the median time patients became wheelchair users because the walking condition was only assessed in the first and the last clinical consultation. However, HAM/TSP patients that presented a clinical progression were in the cohort for at least eight years. Regarding mortality in HAM/TSP patients, it was pointed out that it would be a consequence of complications of the disease itself and co-infections with HCV/HIV^29^. Although there are some case reports of patients with HAM/TSP developing ATL^30^, no large-scale prospective cohort studies have been conducted on patients with HAM/TSP, nor has the risk of ATL transformation been evaluated in these patients. None of the patients included in the present study died of ATL.

Unfortunately, both groups from our HTLV-1 study population were in HW disequilibrium for locus HLA-B, in opposition to the studied 2,624 renal transplant candidates from São Paulo State, Brazil^31^. However, recently a review article discussed the role of HLA-B in 1322 Brazilians compared to different admixed populations worldwide^32^. These authors found that HLA-B gene diversity was higher in admixed and European populations while was lower in African populations. Studying gene diversity in admixed Brazilian population may increase the dataset in others genes banks around the world. As an example, 23 new alleles of the HLA-B gene were exclusively identified in the Brazilian population^32^. The high variability in the HLA-B gene must be studied in light of the role of their alleles in infectious diseases, so common in developing countries. Further studies should address the association of the HLA-B locus with HAM/TSP risk in a large admixed population to increase the likelihood of identifying the disease relationship.

In our cohort, PVL and neurogenic bladder were significantly higher in HAM/TSP than in the AC group, reinforcing what has already been published^33,34^. The close relationship between HTLV-1 infection and uveitis has been identified concerning the ophthalmologic alterations, indicating that HTLV-1 is also highly associated with intraocular inflammatory disorder^35,36^. Some HLA alleles have been related to uveitis. The HLA-A*29 is strongly associated with Birdshot uveitis in individuals of Western-European ancestry, while HLA-B*27 is associated with uveitis, particularly in ankylosing spondylitis^37,38^. In our cohort, ophthalmic manifestations were more frequent in the HAM/TSP group, but it was not possible to correlate it with any HLA due to the small number of affected patients.

Only two studies with small sample sizes were carried out in Brazil to investigate the HLA association with HTLV-1 infection^23,24^. Borducchi et al.^23^ analyzed a small number of HTLV-1-infected patients (n = 71), including AC, ATLL, and HAM/TSP. No statistical difference was determined among these groups because of the small sample size. This study showed a tendency to a lower HLA-A2 frequency in HAM/TSP white patients and an association of HLA-DR11 with HAM/TSP only in the Mestizo patients, in disagreement with our study even after adjustment by ethnicity. Unfortunately, that report did not mention the number of HAM/TSP patients and did not compare their results using multivariable analysis. In another study^24^, only alleles previously associated with protection and disease risk were tested in a small cohort of 84 AC and 9 HAM/TSP patients. They identified that the alleles HLA-C*07 in the absence of -A*02 was associated with an increased risk of disease, and alleles C*08 and B*07 had no association with the development of HAM/TSP. In our study population, the presence or absence of A*02 did not influence the C*07 allele, and C*08 also showed no association with the disease.

In our study, the most frequent allele observed at locus A was A*02 (19.02%, Supplemental Table S1). Still, unlike in the Japanese studies^11,12^, neither A*02 nor the HLA-C*08 alleles were associated with the protection for HAM/TSP development.

Our results showed that the alleles -A*68 and -C*07, had a higher frequency among HAM/TSP patients than in the AC individuals, leading to an increased risk of HAM/TSP. To our knowledge, no previous studies indicated the association of -A*68 with HAM/TSP. However, we should take into account that the -A*68 allele had a higher frequency in our study cohort when compared to the frequency of this allele in the REDOME^25^. Although differences in the frequency could be found among some ethnic groups (greater in black (7.88%) and native Amerindians (7.32%) compared to white (5.66%) ones), the difference observed in patients with HTLV-1 was much more expressive (10.37%), and even greater in HAM/TSP patients (13.29%), suggesting a possible increased risk of this allele for HTLV-1 infection and disease progression in the Brazilian population. Indeed, in an HIV-positive African population, the allele -A*68 was associated with the rapid transmission of HIV in serum discordant heterosexual partners, with 21.3% of frequency^39^ and was associated with a protective role to SARS-COV-2 severe infection and death in a Mexican population, where this allele has a high frequency (12.6% in patients with COVID-19 and 28.6% in the healthy control group)^40^. HLA-A*68 is more frequently identified in South American indigenous populations, such as Argentina, Paraguay, and Chile^41–43^. Besides REDOME data there is a lack of reports describing the frequency of the HLA-A*68 allele in Brazil or in infectious diseases. More studies addressing the role of HLA-A*68 in the Brazilian population in the context of HTLV-1 infection might confirm his association with the augmented risk for infection and disease progression as observed in our study. The -C*07 allele was cited in the Japanese population as a risk factor for HAM/TSP^13^, as well as in our study. Interestedly, in an Argentine population, -C*07 was associated with an increased risk of ATLL but not with HAM/TSP^18^. This allele was underrepresented in AIDS Brazilian patients conferring protection against cytomegalovirus retinitis^44^.

This is the first study that analyzed whether HLA alleles could influence clinical progression in a longitudinal cohort. Patients were clinically followed for 20 years, and their walking clinical progression condition was determined. In our cohort -A*33 and -A*36 were associated with clinical progression and to our knowledge these alleles were not described in other populations. The role of these alleles should be clarified in other populations concerning their physiopathological properties.

There is a clear association between HLA alleles and PVL levels^17–19^. We identified new alleles that were associated with increased PVL in the AC group, carriers of the alleles -C*06, and -DRB1*15, and in the HAM/TSP group, carriers of the alleles -A*30, -A*34, -C*06, -C*17 and -DRB1*09. Although the presence of these alleles influenced an increased PVL, among these alleles, none of them was previously reported. Only B*40 was cited in a Japanese population study^13^, where its distribution was associated with a protective effect. In our cohort HLA-B alleles were in disequilibrium of HW in both cases and control groups.

Further, we analyzed if the presence of the HLA alleles were responsible for the increase in PVL regarding the clinical progression outcome. In the AC group, carriers of the alleles: -C*06, and -DRB1*15 had an increased PVL. In the HAM/TSP group, the alleles -A*30, -A*34, -C*06, -C*17, and -DRB1*09 also influenced PVL and risk for clinical progression. Intriguingly, the alleles that influence PVL and clinical progression in the AC group are not the same as in the HAM/TSP group. Among these alleles, the only correlation with other studies we found in the -DRB1*15 allele, which in the Japanese study had a protective effect^13^ and is cited with a tendency to a higher frequency in HAM/TSP patients than in HCs in a French Afro-Caribbean population^22^, but without association with PVL. It is the first time these associations are described in the Brazilian population.

Moreover, we can highlight that the presence of the HLA-C*06 allele is associated with PVL, clinical progression, and death outcomes for the first time in our population. Studies carried out in ethnically different populations have not demonstrated equanimity in their results regarding risk or protection for the development of HAM/TSP. The host's genetic background must have an important role in this issue.

HTLV-1 has remarkable genetic stability^45^ and two previous reports from Brazilian cities (São Paulo^46^ and Salvador^47^) showed no association with sequenced viral genotype and clinical outcomes. In Brazil, Genotype a (Cosmopolitan) and subgroup A (transcontinental) is the most frequently (> 95%) identified HTLV-1 genotype^48^. None of our enrolled patients was Japanese or their descendant, in which Japanese subgroup B could be identified.

A major limitation of our study is related to the Brazilian genetic background. Brazilian population, as well as all Latin American countries, was composed through centuries of European colonization and the African slave trade. The continuous admixture events among Europeans, Native Americans, and West-Central and Southeast Africans occurred differently in each region of the country, producing a stratified population, even in the current days. Despite our study population showed similar HLA frequencies compared to REDOME (including more than 5 million Brazilians from several regions), it may not reflect the entire genetic diversity of the Brazilian population. Even though, the Brazilian population has a large proportion of European white population^49,50^. Furthermore, Brazil is the large population in Latin America and the fifth-largest in the world, and the Brazilian population is underrepresented in public genomic reference databases^27^. In these databases, 80% of all genetic data is related to the European ancestry population which accounts for only 16% of the world´s population. This gap prevents the discovery of new rare allele variants. Few studies reported the ancestry background of the general Brazilian population^51^ and studies associating ancestry analysis and HLA frequencies could better relate the role of HLA polymorphisms in the HTLV-1 infection and its medical importance. Furthermore, the study was conducted with clinical data collected retrospectively in a convenience sample. It was impossible to match patients with similar and long clinical follow-up times and with similar disease stages. Another limitation of this study was the reduction of the HLA typing to the level of allelic group. Although we have used in part of the typing a medium-level resolution that could result in better discrimination of the HLA alleles of risk and susceptibility, we were also limited by the HLA frequencies that tend to be small and implying having a larger cohort to have statistical differences. Nonetheless, we are not aware that allelic level should be the final goal for HLA studies, as in many diseases that initially were associated with an antigen (allele group level) that with further studies have pointed out that only some alleles of the group were in association with the disease, as for spondyloarthropathies with HLA-B*27^52^ or rheumatic arthritis HLA-DRB*04^53^. Our results show an association between HLA frequencies and HAM/TSP risk, PVL, disease progression, and death. Because of the low number of participants presenting some HLA alleles, the results observed in this work have to be confirmed in other populations. Nevertheless, in light of our acknowledgment, this is the study involving the highest number of participants associating HLA alleles and HTLV-1 infection in the Brazilian population. Future studies, designed with an even greater number of patients, with a long and similar clinical follow-up period, and which include patients in the early stages of the disease, may increase the strength of comparisons between some HLAs regarding their role in HTLV-1 infection in the Brazilian population, reinforcing our findings.

The HLA system is vital in response to infections. In the case of HTLV-1 infection, where a minority of individuals carrying the virus will develop the neurological disease, the genetic determinants could be seen as an interplay between different immunological events. However, more studies should be conducted to monitor the HLA profile in AC patients at risk of developing the disease in longitudinal follow-up.

## Conclusions

In this study, we investigated the influence of HLA polymorphisms on the susceptibility for the development of HAM/TSP and clinical progression in a longitudinal cohort of an admixed Brazilian population. We found that HLA-A*68 and -C*07 carriers presented an augmented risk for HAM/TSP development in HTLV-1-infected individuals. In addition, -C*12, -C*14, and -DRB1*08 were associated with an augmented risk of death in HAM/TSP individuals. In contrast, the alleles HLA-A*33, and -A*36 were related to protection against disease progression in these individuals. We also showed that asymptomatic -C*06 and -DRB1*15 carriers have increased proviral load, while -A*30, -A*34, -C*06, -C*17, and -DRB1*09 alleles were associated with this characteristic on HAM/TSP patients. We also demonstrated that patients with high PVL and carrying these alleles presented disease clinical progression, while the presence of HLA-A*30 in these patients was associated with death. This is the first study to evaluate the influence of HLA alleles on the risk for HAM/TSP development as well as in clinical progression and death, in an admixed Brazilian population. Brazil has one of the highest rates of HTLV-1 infection, and studies addressing genetic factors associated with HAM/TSP risk could prevent disease worsening through earlier medical care.

## Methods

### Patients and samples

The Laboratory for Clinical Research in Neuroinfections at the Evandro Chagas National Institute of Infectious Diseases (Lapclin-Neuro), FIOCRUZ (INI/Fiocruz), Rio de Janeiro, RJ, Brazil, is a reference center for diagnostic and clinical follow-up of asymptomatic carriers and patients with neurological manifestations of HTLV-1 infection. Since 1999 more than 1000 AC and neurological patients, the majority of them with HAM/TSP, have been investigated and followed up in a longitudinal cohort by a team of neurologists. According to their clinical visit, HTLV-1 positive and genetically unrelated patients were randomly and consecutively included from the longitudinal study cohort. The Research Ethics Committee of the INI/FIOCRUZ (CAAE-0012.0.009.000-08) approved this study, and all subjects provided written informed consent. Volunteers were consecutively followed from 1999 to 2020 in a longitudinal cohort study from eight to 22 years. A sex, age, and ethnicity-matched unrelated case–control study was then designed to identify HLA's influence on the disease disability status, PVL, disease progression, and death outcomes. All clinical data were collected retrospectively, using a convenience sample provided by the cohort follow-up. A comprehensive medical history relating to neurological illnesses was taken at the time of inclusion in the cohort and annually for the AC patients and biannually for the HAM/TSP patients.

The clinical assessment and classification of the neurological cases followed the World Health Organization guidelines and diagnostic criteria for HAM/TSP^54^. Patients’ inclusion criteria were defined by positive serology for HTLV-1 in the blood (for AC) and blood and cerebrospinal fluid (CSF) for neurological patients (HAM/TSP and other neurological findings). The screening for HTLV-1/2 infection was performed throughout the serological test using a Recombinant ELISA HTLV1/2 kit from Wiener (Rosario, Argentina), and confirmation of HTLV-1 infection through western blot analysis (Cambridge Biotech, Worcester, MA, USA). Patients with HTLV-2 infection were not enrolled in the study*.* Individuals were divided into two groups: individuals with HAM/TSP, and other isolated neurological findings such as isolated neurogenic bladder, and HTLV-1 asymptomatic carriers (AC). The HAM/TSP participants were classified according to their disability status using the IPEC Clinical Disability Scale (IPEC-CDS) as described elsewhere^55^. The IPEC disability scale is a numerical scale ranging from 0 (no disability) to 29 (worse neurological status). This scale is a composite of motor, pain, sensory, and sphincter subscores^55^.

Clinical progression was determined by comparing patients' gait performance according to the IPEC-CDS motor subscore at the first clinical visit, when patients were included in the cohort, with the same score obtained at the last clinical visit. Any worsening in the IPEC-CDS motor score was considered disease progression, regardless of the initial score assigned to patients at the time of study enrollment. We also did not consider the degree of progression, meaning that patients who progressed to two or more levels on the motor scale were not separated from those who progressed to only one level.

A survival curve was generated to analyze the longitudinal clinical progression in HAM/TSP patients, using each of their initial clinical classifications regarding the ability to walk (walk without assistance, with unilateral or bilateral support, and the need for a wheelchair). A change in the motor scale of worsening prognosis was considered a sign of disease progression.

### HLA genotyping

Peripheral blood (5 mL) was collected in EDTA tubes from all individuals, and DNA was extracted using the Puregene commercial kit (Gentra Systems Inc., Minneapolis, MN, USA), following the manufacturer’s instructions.

First, the HTLV-1 cohort was HLA genotyped through an in-house PCR-SSP (polymerase chain reaction-sequence-specific primer) from 1999 to 2007 (n = 273), and throughout PCR-SSO (polymerase chain reaction with specific oligonucleotide probes) technique (n = 102).

The HLA class I loci (-A, -B, and -C) genotyping was performed in an in-house PCR-SSP according to the methodology established by the Organ Transplant Laboratory from Oxford University^56^. Primers were donated by the Department of Immunology at the St Mary’s School of Medicine, Imperial College, London, UK, and the PCR-SSO technique, using DNA LABType® trading system (One Lambda Inc., Canoga Park, CA, USA) was performed, according to manufacturer`s instructions, that provides SSO probes for the sequence-specific oligonucleotide linked to fluorescent microspheres for the identification of HLA alleles in genomic DNA samples.

### Proviral load quantification

The HTLV-1 PVL DNA was measured by real-time PCR assay (SmartCycle II; Cepheid) using the TaqMan system (Applied Biosystems) to amplify a 159 bp fragment of tax gene with SK43 and SK44 primers. A standard curve was generated using the β-globin gene as a reference. DNA from the TARL-2 cell line, which contains a single copy of the provirus HTLV-1, was used to establish the standard curve for tax gene quantification. The proviral load (PVL) in 10^4^ cells was calculated by the following equation: [(copy number of tax gene)/ (copy number of β-globin gene/2)] × 10,000^33^. The mean of proviral load measures during the follow-up was used to determine the proviral load of each patient.

### Statistical analysis

A descriptive analysis of clinical and demographic characteristics was performed. Outcomes (clinical status—HAM/TSP or AC, disease progression, and death) and alleles (HLA-A, -B, -C, and -DR) were associated using absolute and relative frequencies or mean and standard deviation. Hardy–Weinberg equilibrium was calculated by Exact test using a Markov chain to define the equilibrium in the allelic distribution in HLA-A, -B, -C, and -DR locus between HAM/TSP and AC patients. Prism 7 was used to determine a survival curve to analyze the walking ability's clinical progression. The Mann–Whitney U test evaluated quantitative variables, whereas the univariate Odds Ratio (OR) was used for categorical variables. We used a multivariate generalized linear Binomial model to estimate OR and Ci = Is. Linear models were tested to estimate the average difference in PVL among alleles and outcomes. A *p*-value < 0.05 was considered significant. All analysis was performed using R software version 3.6.3.

### Ethics statement

All research was performed following relevant guidelines/regulations described by the policies of the Nature Portfolio journals, and the Declaration of Helsinki. This study was approved by the Research Ethics Committee of the INI/FIOCRUZ (CAAE-0012.0.009.000-08) and all participants provided written informed consent.

## Supplementary Information

Supplementary Information 1.

Supplementary Information 2.

Supplementary Information 3.

## Data Availability

All data generated or analyzed during this study are included in this published article [and its supplemental information files].

## References

[1] Uchiyama T, Yodoi J, Sagawa K, Takatsuki K, Uchino H (1977). Adult T-cell leukemia: clinical and hematologic features of 16 cases. Blood.

[2] Gessain A, Barin F, Vernant JC, Gout O, Maurs L, Calender A (1985). Antibodies to human T-lymphotropic virus type-I in patients with tropical spastic paraparesis. Lancet.

[3] Osame M, Usuku K, Izumo S, Ijichi N, Amitani H, Igata A (1986). HTLV-I associated myelopathy, a new clinical entity. Lancet.

[4] Gessain A, Cassar O (2012). Epidemiological aspects and world distribution of HTLV-1 infection. Front. Microbiol..

[5] Bangham CRM, Araujo A, Yamano Y, Taylor GP (2015). HTLV-1-associated myelopathy/tropical spastic paraparesis. Nat. Rev. Dis. Primers..

[6] Yamano Y, Sato T (2012). Clinical pathophysiology of human T-lymphotropic virus-type 1-associated myelopathy/tropical spastic paraparesis. Front. Microbiol..

[7] Castro NM, Rodrigues W, Freitas DM, Muniz A, Oliveira P, Carvalho EM (2007). Urinary symptoms associated with human T-cell lymphotropic virus type I infection: Evidence of urinary manifestations in large group of HTLV-I carriers. Urology.

[8] Rathsam-Pinheiro RH, Boa-Sorte N, Grassi MFR, Copello ÚC, Rios KTSG, Araújo T (2019). Revisiting Keratoconjunctivitis sicca associated with Human T-Cell Lymphotropic Virus Type 1: prevalence, clinical aspects and proviral load. Braz. J. Infect. Dis..

[9] Ijichi S, Izumo S, Eiraku N, Machigashira K, Kubota R, Nagai M (1993). An autoaggressive process against bystander tissues in HTLV-I-infected individuals: a possible pathomechanism of HAM/TSP. Med. Hypotheses..

[10] Nishimura Y, Okubo R, Minato S, Itoyama Y, Goto I, Mori M (1991). A possible association between HLA and HTLV-I-associated myelopathy (HAM) in Japanese. Tissue Antigens.

[11] Jeffery KJ, Usuku K, Hall SE, Matsumoto W, Taylor GP, Procter J (1999). HLA alleles determine human T-lymphotropic virus-I (HTLV-I) proviral load and the risk of HTLV-I-associated myelopathy. Proc. Natl. Acad. Sci. U S A..

[12] Jeffery KJ, Siddiqui AA, Bunce M, Lloyd AL, Vine AM, Witkover AD (2000). The influence of HLA class I alleles and heterozygosity on the outcome of human T cell lymphotropic virus type I infection. J. Immunol..

[13] Penova M, Kawaguchi S, Yasunaga J-I, Kawaguchi T, Sato T, Takahashi M (2021). Genome wide association study of HTLV-1-associated myelopathy/tropical spastic paraparesis in the Japanese population. Proc. Natl. Acad. Sci. U S A..

[14] Kitze B, Usuku K, Yamano Y, Yashiki S, Nakamura M, Fujiyoshi T (1998). Human CD4+ T lymphocytes recognize a highly conserved epitope of human T lymphotropic virus type 1 (HTLV-1) env gp21 restricted by HLA DRB1*0101. Clin. Exp. Immunol..

[15] Sabouri AH, Saito M, Usuku K, Bajestan SN, Mahmoudi M, Forughipour M (2005). Differences in viral and host genetic risk factors for development of human T-cell lymphotropic virus type 1 (HTLV-1)-associated myelopathy/tropical spastic paraparesis between Iranian and Japanese HTLV-1-infected individuals. J. Gen. Virol..

[16] Rafatpanah H, Pravica V, Faridhosseini R, Tabatabaei A, Ollier W, Poulton K (2007). Association between HLA-DRB1*01 and HLA-Cw*08 and outcome following HTLV-I infection. Iran J. Immunol..

[17] Treviño A, Vicario JL, Lopez M, Parra P, Benito R, Ortiz Lejarazu R (2013). Association between HLA alleles and HAM/TSP in individuals infected with HTLV-1. J. Neurol..

[18] Benencio P, Fraile Gonzalez SA, Ducasa N, Page K, Berini CA, Biglione MM (2020). HLA-B*35 as a new marker for susceptibility to human T-cell lymphotropic virus type 1 (HTLV-1) Associated Myelopathy/Tropical Spastic Paraparesis (HAM/TSP) in patients living in Argentina. Retrovirology.

[19] Taghaddosi M, Rezaee SAR, Rafatpanah H, Rajaei T, Farid Hosseini R, Narges V (2013). Association between HLA Class I Alleles and Proviral Load in HTLV-I associated myelopathy/tropical spastic paraperesis (HAM/TSP) patients in Iranian population. Iran. J. Basic Med. Sci..

[20] Tarokhian H, Taghadosi M, Rafatpanah H, Rajaei T, Azarpazhooh MR, Valizadeh N (2017). The effect of HTLV-1 virulence factors (HBZ, Tax, proviral load), HLA class I and plasma neopterin on manifestation of HTLV-1 associated myelopathy tropical spastic paraparesis. Virus Res..

[21] Talledo M, López G, Huyghe JR, Verdonck K, Adaui V, González E (2010). Evaluation of host genetic and viral factors as surrogate markers for HTLV-1-associated myelopathy/tropical spastic paraparesis in Peruvian HTLV-1-infected patients. J. Med. Virol..

[22] Deschamps R, Béra O, Belrose G, Lezin A, Bellance R, Signate A (2010). Absence of consistent association between human leukocyte antigen-I and -II alleles and human T-lymphotropic virus type 1 (HTLV-1)-associated myelopathy/tropical spastic paraparesis risk in an HTLV-1 French Afro-Caribbean population. Int. J. Infect. Dis..

[23] Borducchi DM, Gerbase-DeLima M, Morgun A, Shulzhenko N, Pombo-de-Oliveira MS, Kerbauy J (2003). Human leucocyte antigen and human T-cell lymphotropic virus type 1 associated diseases in Brazil. Br. J. Haematol..

[24] Catalan-Soares BC, Carneiro-Proietti ABF, Da Fonseca FG, Correa-Oliveira R, Peralva-Lima D, Portela R (2009). HLA class I alleles in HTLV-1-associated myelopathy and asymptomatic carriers from the Brazilian cohort GIPH. Med. Microbiol. Immunol..

[25] Perfil Genômico do REDOME/REREME [Internet]. Rede Brasil de Imunogenética. [cited 2022 Feb 17]. Available from: http://imunogenetica.org/resultados/perfil-genomico-do-redome-rereme/.

[26] Saito M (2019). Association between HTLV-1 genotypes and risk of HAM/TSP. Front. Microbiol..

[27] Rocha CS, Secolin R, Rodrigues MR, Carvalho BS, Lopes-Cendes I (2020). The Brazilian Initiative on Precision Medicine (BIPMed): Fostering genomic data-sharing of underrepresented populations. NPJ Genom. Med..

[28] Araujo AQC (2019). Neurologic complications of HTLV-1: A review. Rev. Bras. Neurol..

[29] Marcusso RMN, Van Weyenbergh J, de Moura JVL, Dahy FE (2019). Dichotomy in fatal outcomes in a large cohort of people living with HTLV-1 in São Paulo Brazil. Pathogens.

[30] Martin F, Fedina A, Youshya S, Taylor GP (2010). A 15-year prospective longitudinal study of disease progression in patients with HTLV-1 associated myelopathy in the UK. J. Neurol. Neurosurg. Psychiatry..

[31] Ravazzi-Gauch C, Bajay MM, Caldas HC, Abbud-Filho M (2016). HLA-A, -B, and -DRB1 allele and haplotype diversity in a cohort of Brazilian renal transplant candidates. Hum. Immunol..

[32] Silva, N.D.S.B., Souza, A.S., Andrade, H.S., Pereira, R.N., Castro, C.F.B., & Vince, N., *et al.* Immunogenetics of HLA-B: SNP, allele, and haplotype diversity in populations from different continents and ancestry backgrounds. *HLA* (2023). 10.1111/tan.15043 37005006

[33] Silva MTT, Harab RC, Leite AC, Schor D, Araújo A, Andrada-Serpa MJ (2007). Human T lymphotropic virus type 1 (HTLV-1) proviral load in asymptomatic carriers, HTLV-1-associated myelopathy/tropical spastic paraparesis, and other neurological abnormalities associated with HTLV-1 infection. Clin. Infect. Dis..

[34] Silva MT, Coutinho F, Leite AC, Harab RC, Araújo A, Andrada-Serpa MJ (2009). Isolated bladder dysfunction in human T lymphotropic virus type 1 infection. Clin. Infect. Dis..

[35] Mochizuki M, Watanabe T, Yamaguchi K, Tajima K, Yoshimura K, Nakashima S (1992). Uveitis associated with human T lymphotropic virus type I: seroepidemiologic, clinical, and virologic studies. J. Infect. Dis..

[36] Kamoi K (2020). HTLV-1 in ophthalmology. Front. Microbiol..

[37] Kuiper JJW, Venema WJ (2020). HLA-A29 and Birdshot Uveitis: Further Down The Rabbit Hole. Front. Immunol..

[38] İnanç M, Şimşek M, Çakar Özdal MP (2019). Etiological and Clinical Characteristics of HLA-B27-associated Uveitis in a Tertiary Referral Center. Turk. J. Ophthalmol..

[39] Song W, He D, Brill I, Malhotra R, Mulenga J, Allen S (2011). Disparate associations of HLA class I markers with HIV-1 acquisition and control of viremia in an African population. PLoS ONE.

[40] Hernández-Doño S, Sánchez-González RA, Trujillo-Vizuet MG, Zamudio-Castellanos FY, García-Silva R, Bulos-Rodríguez P (2022). Protective HLA alleles against severe COVID-19: HLA-A*68 as an ancestral protection allele in Tapachula-Chiapas Mexico. Clin. Immunol..

[41] Rey D, Parga-Lozano C, Moscoso J, Areces C, Enriquez-de-Salamanca M, Fernández-Honrado M (2013). HLA genetic profile of Mapuche (Araucanian) Amerindians from Chile. Mol. Biol. Rep..

[42] Layrisse Z, Guedez Y, Domínguez E, Paz N, Montagnani S, Matos M (2001). Extended HLA haplotypes in a Carib Amerindian population: the Yucpa of the Perija Range. Hum. Immunol..

[43] Single RM, Meyer D, Nunes K, Francisco RS, Hünemeier T, Maiers M (2020). Demographic history and selection at HLA loci in Native Americans. PLoS ONE.

[44] Biberg-Salum TG, Veronese Rodrigues ML, Fernandes APM, Deghaide NHS, Paula JS, Castelli EC (2018). HLA-C alleles and cytomegalovirus retinitis in Brazilian patients with AIDS. J. Ophthalmol..

[45] Gessain A, Gallo RC, Franchini G (1992). Low degree of human T-cell leukemia/lymphoma virus type I genetic drift in vivo as a means of monitoring viral transmission and movement of ancient human populations. J. Virol..

[46] Pessôa R, Watanabe JT, Nukui Y, Pereira J, Casseb J, Kasseb J (2014). Molecular characterization of human T-cell lymphotropic virus type 1 full and partial genomes by Illumina massively parallel sequencing technology. PLoS ONE.

[47] Araújo THA, Barreto FK, Menezes ADL, Lima CPS, Oliveira RS, Lemos PS (2020). Complete genome sequence of human T-cell lymphotropic type 1 from patients with different clinical profiles, including infective dermatitis. Infect. Genet. Evol..

[48] Afonso PV, Cassar O, Gessain A (2019). Molecular epidemiology, genetic variability and evolution of HTLV-1 with special emphasis on African genotypes. Retrovirology.

[49] Kehdy FSG, Gouveia MH, Machado M, Magalhães WCS, Horimoto AR, Horta BL (2015). Origin and dynamics of admixture in Brazilians and its effect on the pattern of deleterious mutations. Proc. Natl. Acad. Sci. U S A..

[50] Secolin R, Mas-Sandoval A, Arauna LR, Torres FR, de Araujo TK, Santos ML (2019). Distribution of local ancestry and evidence of adaptation in admixed populations. Sci. Rep..

[51] De Oliveira TC, Secolin R, Lopes-Cendes I (2023). A review of ancestrality and admixture in Latin America and the caribbean focusing on native American and African descendant populations. Front Genet..

[52] Fahed H, Mauro D, Ciccia F, Ziade NR (2020). What does human leukocyte antigen B27 have to do with spondyloarthritis?. Rheum Dis Clin North Am..

[53] Becart S, Whittington KB, Prislovsky A, Rao NL, Rosloniec EF (2021). The role of posttranslational modifications in generating neo-epitopes that bind to rheumatoid arthritis-associated HLA-DR alleles and promote autoimmune T cell responses. PLoS ONE.

[54] Osame, M. Review of WHO Kagoshima meeting and diagnostic guidelines for HAM/TSP. Human Retrovirology: HTLV. Raven; p. 191–7 (1990).

[55] Lima MA, Bica RBS, Araújo AQC (2005). Gender influence on the progression of HTLV-I associated myelopathy/tropical spastic paraparesis. J. Neurol. Neurosurg. Psychiatry..

[56] Bunce M, O’Neill CM, Barnardo MC, Krausa P, Browning MJ, Morris PJ (1995). Phototyping: Comprehensive DNA typing for HLA-A, B, C, DRB1, DRB3, DRB4, DRB5 & DQB1 by PCR with 144 primer mixes utilizing sequence-specific primers (PCR-SSP). Tissue Antigens.

