# Supplementary material for: Putative role of HLA polymorphism among a Brazilian HTLV-1-associated myelopathy/tropical spastic paraparesis (HAM/TSP) population
Source: Sci Rep. 2023 May 11;13:7659. doi: 10.1038/s41598-023-34757-w (PMC10173239; doi:10.1038/s41598-023-34757-w)
Supplement: Supplementary file 1 — Supplementary Information 1. [file 41598_2023_34757_MOESM1_ESM.docx]

| Supplemental Table S1. Frequency of HLA-A, -B, -C and -DRB1 loci in HAM/TSP and AC Brazilian patients | | | | | | | |
| --- | --- | --- | --- | --- | --- | --- | --- |
|  | **HAM-TSP (n=210)** | **AC (n=165)** | **OR (95%CI)** | **P-value** | **OR-A (95%CI)*** | **P-value*** |  |
| **A Locus n (%)** |  |  |  |  |  |  |  |
| **-A*01** | 26 (6.3) | 21 (6.7) | 0.94 (0.52-1.71) | 0.85 | 0.97 (0.52-1.79) | 0.92 |  |
| **-A*02** | 75 (18.2) | 64 (20.3) | 0.87 (0.6-1.27) | 0.47 | 0.84 (0.57-1.23) | 0.37 |  |
| **-A*03** | 37 (9) | 28 (8.9) | 1.01 (0.6-1.69) | 0.97 | 1.17 (0.68-1.99) | 0.57 |  |
| **-A*11** | 31 (7.5) | 18 (5.7) | 1.34 (0.74-2.45) | 0.34 | 1.38 (0.73-2.61) | 0.32 |  |
| **-A*23** | 27 (6.6) | 20 (6.3) | 1.03 (0.57-1.88) | 0.91 | 0.93 (0.5-1.75) | 0.82 |  |
| **-A*24** | 41 (10) | 37 (11.7) | 0.83 (0.52-1.33) | 0.44 | 0.83 (0.51-1.37) | 0.47 |  |
| **-A*25** | 1 (0.2) | 0 (0) | - | - | - | - |  |
| **-A*26** | 17 (4.1) | 8 (2.5) | 1.65 (0.7-3.88) | 0.25 | 1.65 (0.67-4.09) | 0.28 |  |
| **-A*29** | 13 (3.2) | 14 (4.4) | 0.7 (0.32-1.51) | 0.36 | 0.6 (0.27-1.35) | 0.22 |  |
| **-A*30** | 23 (5.6) | 24 (7.6) | 0.72 (0.4-1.3) | 0.27 | 0.74 (0.4-1.35) | 0.32 |  |
| **-A*31** | 16 (3.9) | 12 (3.8) | 1.02 (0.48-2.19) | 0.96 | 1 (0.45-2.22) | 1.00 |  |
| **-A*32** | 10 (2.4) | 8 (2.5) | 0.95 (0.37-2.45) | 0.92 | 0.89 (0.34-2.38) | 0.82 |  |
| **-A*33** | 14 (3.4) | 11 (3.5) | 0.97 (0.44-2.17) | 0.95 | 0.82 (0.36-1.89) | 0.64 |  |
| **-A*34** | 4 (1) | 6 (1.9) | 0.5 (0.14-1.8) | 0.29 | 0.54 (0.15-1.95) | 0.35 |  |
| **-A*36** | 4 (1) | 3 (1) | 1.02 (0.23-4.59) | 0.98 | 1.43 (0.3-6.85) | 0.65 |  |
| **-A*66** | 7 (1.7) | 8 (2.5) | 0.66 (0.24-1.85) | 0.43 | 0.8 (0.27-2.34) | 0.68 |  |
| **-A*68** | 55 (13.3) | 23 (7.3) | 1.96 (1.17-3.26) | 0.01 | 2.03 (1.19-3.46) | 0.01 |  |
| **-A*74** | 10 (2.4) | 10 (3.2) | 0.76 (0.31-1.85) | 0.54 | 0.76 (0.3-1.92) | 0.56 |  |
| **-A*80** | 1 (0.2) | 0 (0) | - | - | - | - |  |
|  |  |  |  |  |  |  |  |
| **B Locus n (%)** |  |  |  |  |  |  |  |
| **-B*07** | 13 (4.6) | 13 (4.2) | 1.09 (0.49-2.38) | 0.84 | 1.03 (0.46-2.31) | 0.93 |  |
| **-B*08** | 16 (5.7) | 10 (3.3) | 1.77 (0.79-3.98) | 0.16 | 1.81 (0.79-4.15) | 0.16 |  |
| **-B*13** | 2 (0.7) | 3 (1) | 0.72 (0.12-4.33) | 0.72 | 0.77 (0.12-4.91) | 0.78 |  |
| **-B*14** | 10 (3.5) | 11 (3.6) | 0.98 (0.41-2.35) | 0.97 | 0.85 (0.35-2.09) | 0.73 |  |
| **-B*15** | 31 (11) | 33 (10.8) | 1.02 (0.61-1.71) | 0.95 | 1.02 (0.6-1.75) | 0.93 |  |
| **-B*18** | 16 (5.7) | 9 (2.9) | 1.98 (0.86-4.55) | 0.11 | 2.02 (0.86-4.76) | 0.11 |  |
| **-B*27** | 8 (2.8) | 5 (1.6) | 1.75 (0.57-5.42) | 0.33 | 1.67 (0.51-5.49) | 0.40 |  |
| **-B*35** | 27 (9.5) | 39 (12.7) | 0.72 (0.43-1.21) | 0.22 | 0.71 (0.42-1.22) | 0.22 |  |
| **-B*37** | 0 (0) | 5 (1.6) | - | - | - | - |  |
| **-B*38** | 10 (3.5) | 5 (1.6) | 2.21 (0.74-6.53) | 0.15 | 2.82 (0.91-8.75) | 0.07 |  |
| **-B*39** | 4 (1.4) | 2 (0.7) | 2.18 (0.4-11.99) | 0.37 | 1.58 (0.28-8.94) | 0.61 |  |
| **-B*40** | 13 (4.6) | 17 (5.6) | 0.82 (0.39-1.72) | 0.60 | 0.96 (0.45-2.04) | 0.91 |  |
| **-B*41** | 4 (1.4) | 3 (1) | 1.45 (0.32-6.53) | 0.63 | 1.31 (0.28-6.16) | 0.73 |  |
| **-B*42** | 8 (2.8) | 11 (3.6) | 0.78 (0.31-1.97) | 0.60 | 0.88 (0.34-2.28) | 0.80 |  |
| **-B*44** | 18 (6.4) | 30 (9.8) | 0.62 (0.34-1.15) | 0.13 | 0.61 (0.32-1.14) | 0.12 |  |
| **-B*45** | 6 (2.1) | 3 (1) | 2.19 (0.54-8.83) | 0.27 | 2.2 (0.53-9.04) | 0.28 |  |
| **-B*47** | 0 (0) | 2 (0.7) | - | - | - | - |  |
| **-B*48** | 8 (2.8) | 5 (1.6) | 1.75 (0.57-5.42) | 0.33 | 1.99 (0.62-6.38) | 0.24 |  |
| **-B*49** | 5 (1.8) | 6 (2) | 0.9 (0.27-2.98) | 0.86 | 0.67 (0.19-2.39) | 0.54 |  |
| **-B*50** | 8 (2.8) | 8 (2.6) | 1.08 (0.4-2.93) | 0.87 | 1 (0.36-2.79) | 0.99 |  |
| **-B*51** | 18 (6.4) | 19 (6.2) | 1.03 (0.53-2) | 0.94 | 0.85 (0.43-1.68) | 0.63 |  |
| **-B*52** | 6 (2.1) | 7 (2.3) | 0.93 (0.31-2.79) | 0.89 | 0.7 (0.23-2.17) | 0.54 |  |
| **-B*53** | 15 (5.3) | 17 (5.6) | 0.95 (0.47-1.94) | 0.89 | 1.25 (0.59-2.66) | 0.55 |  |
| **-B*55** | 0 (0) | 4 (1.3) | - | - | - | - |  |
| **-B*56** | 1 (0.4) | 0 (0) | - | - | - | - |  |
| **-B*57** | 13 (4.6) | 15 (4.9) | 0.93 (0.44-2) | 0.86 | 1.01 (0.47-2.2) | 0.97 |  |
| **-B*58** | 20 (7.1) | 15 (4.9) | 1.48 (0.74-2.94) | 0.27 | 1.61 (0.79-3.3) | 0.19 |  |
| **-B*81** | 1 (0.4) | 7 (2.3) | 0.15 (0.02-1.24) | 0.08 | 0.15 (0.02-1.26) | 0.08 |  |
| **-B*82** | 2 (0.7) | 2 (0.7) | 1.08 (0.15-7.73) | 0.94 | 0.77 (0.1-5.7) | 0.80 |  |
|  |  |  |  |  |  |  |  |
| **C Locus n (%)** |  |  |  |  |  |  |  |
| **-C*01** | 4 (1.4) | 4 (1.4) | 1.06 (0.26-4.28) | 0.94 | 0.94 (0.22-4.01) | 0.93 |  |
| **-C*02** | 22 (8) | 30 (10.3) | 0.76 (0.42-1.35) | 0.34 | 0.78 (0.43-1.41) | 0.41 |  |
| **-C*03** | 29 (10.5) | 33 (11.3) | 0.92 (0.54-1.56) | 0.76 | 0.86 (0.5-1.48) | 0.58 |  |
| **-C*04** | 46 (16.7) | 66 (22.6) | 0.68 (0.45-1.04) | 0.08 | 0.69 (0.45-1.06) | 0.09 |  |
| **-C*05** | 14 (5.1) | 9 (3.1) | 1.68 (0.72-3.95) | 0.23 | 1.73 (0.72-4.17) | 0.22 |  |
| **-C*06** | 26 (9.4) | 26 (8.9) | 1.06 (0.6-1.88) | 0.83 | 0.99 (0.55-1.77) | 0.96 |  |
| **-C*07** | 63 (22.8) | 47 (16.1) | 1.54 (1.01-2.35) | 0.04 | 1.61 (1.04-2.48) | 0.03 |  |
| **-C*08** | 14 (5.1) | 17 (5.8) | 0.86 (0.42-1.79) | 0.69 | 0.92 (0.43-1.94) | 0.82 |  |
| **-C*12** | 16 (5.8) | 19 (6.5) | 0.88 (0.45-1.76) | 0.73 | 0.92 (0.45-1.89) | 0.83 |  |
| **-C*14** | 8 (2.9) | 7 (2.4) | 1.22 (0.43-3.4) | 0.71 | 1.03 (0.36-2.96) | 0.96 |  |
| **-C*15** | 9 (3.3) | 11 (3.8) | 0.86 (0.35-2.11) | 0.74 | 0.86 (0.34-2.18) | 0.75 |  |
| **-C*16** | 20 (7.2) | 15 (5.1) | 1.44 (0.72-2.88) | 0.30 | 1.45 (0.71-2.93) | 0.31 |  |
| **-C*17** | 5 (1.8) | 6 (2.1) | 0.88 (0.27-2.92) | 0.83 | 0.87 (0.25-2.95) | 0.82 |  |
| **-C*18** | 0 (0) | 2 (0.7) | - | - | - | - |  |
|  |  |  |  |  |  |  |  |
| **DRB1 Locus n (%)** | |  |  |  |  |  |  |
| **-DRB1*01** | 30 (11.2) | 32 (10.6) | 1.06 (0.63-1.8) | 0.82 | 1.02 (0.59-1.75) | 0.94 |  |
| **-DRB1*03** | 29 (10.8) | 20 (6.6) | 1.71 (0.94-3.1) | 0.08 | 1.68 (0.91-3.09) | 0.10 |  |
| **-DRB1*04** | 30 (11.2) | 37 (12.3) | 0.9 (0.54-1.51) | 0.70 | 0.86 (0.51-1.46) | 0.57 |  |
| **-DRB1*07** | 30 (11.2) | 45 (14.9) | 0.72 (0.44-1.18) | 0.19 | 0.72 (0.43-1.19) | 0.20 |  |
| **-DRB1*08** | 12 (4.5) | 16 (5.3) | 0.84 (0.39-1.8) | 0.65 | 0.81 (0.37-1.77) | 0.60 |  |
| **-DRB1*09** | 9 (3.4) | 7 (2.3) | 1.46 (0.54-3.99) | 0.46 | 1.41 (0.51-3.92) | 0.51 |  |
| -**DRB1*10** | 11 (4.1) | 5 (1.7) | 2.54 (0.87-7.41) | 0.09 | 2.98 (0.99-8.96) | 0.05 |  |
| **-DRB1*11** | 40 (14.9) | 45 (14.9) | 1 (0.63-1.59) | 0.99 | 1.06 (0.66-1.69) | 0.82 |  |
| **-DRB1*12** | 5 (1.9) | 6 (2) | 0.94 (0.28-3.11) | 0.92 | 0.94 (0.28-3.19) | 0.92 |  |
| **-DRB1*13** | 31 (11.6) | 48 (15.9) | 0.69 (0.43-1.12) | 0.14 | 0.71 (0.43-1.16) | 0.17 |  |
| **-DRB1*14** | 8 (3) | 10 (3.3) | 0.9 (0.35-2.31) | 0.82 | 0.85 (0.33-2.22) | 0.74 |  |
| **-DRB1*15** | 22 (8.2) | 23 (7.6) | 1.08 (0.59-1.99) | 0.79 | 1.14 (0.61-2.13) | 0.68 |  |
| **-DRB1*16** | 11 (4.1) | 8 (2.6) | 1.57 (0.62-3.97) | 0.34 | 1.51 (0.58-3.88) | 0.40 |  |
| AC= asymptomatic control, HAM/TSP= Human T cell lymphotropic virus-associated myelopathy/tropical spastic paraparesis, OR= odds ratio, 95% confidence interval, OR-A=odds ratio adjusted, * *p* values adjusted by age, sex, ethnicity and time of clinical follow-up. | | | | | | |  |

| Supplemental Table S6. Frequency of HLA-A, -B, -C and -DRB1 loci in HAM/TSP patients related to progression outcome. | | | | | | | |
| --- | --- | --- | --- | --- | --- | --- | --- |
|  | **Progression** | |  |  |  |  |  |
|  | **No (n=34)** | **Yes (n=148)** | **OR (95%CI)** | **P-value** | **A-OR (95%CI)*** | **P-value*** |  |
| **A Locus n (%)** |  |  |  |  |  |  |  |
| **-A*01** | 6 (9) | 19 (6.6) | 0.72 (0.27-1.87) | 0.49 | 0.7 (0.26-1.93) | 0.49 |  |
| **-A*02** | 9 (13.4) | 58 (20.1) | 1.62 (0.76-3.46) | 0.21 | 1.75 (0.8-3.82) | 0.16 |  |
| **-A*03** | 8 (11.9) | 25 (8.7) | 0.7 (0.3-1.63) | 0.4 | 0.66 (0.27-1.6) | 0.35 |  |
| **-A*11** | 4 (6) | 22 (7.6) | 1.3 (0.43-3.9) | 0.64 | 1.63 (0.51-5.17) | 0.41 |  |
| **-A*23** | 1 (1.5) | 23 (8) | 5.71 (0.76-43.01) | 0.09 | 5.87 (0.76-45.16) | 0.09 |  |
| **-A*24** | 5 (7.5) | 29 (10) | 1.38 (0.51-3.72) | 0.52 | 1.18 (0.42-3.27) | 0.75 |  |
| **-A*25** | 1 (1.5) | 0 (0) | - | - | - | - |  |
| **-A*26** | 4 (6) | 9 (3.1) | 0.51 (0.15-1.7) | 0.27 | 0.62 (0.17-2.3) | 0.47 |  |
| **-A*29** | 2 (3) | 9 (3.1) | 1.04 (0.22-4.95) | 0.96 | 1.11 (0.22-5.51) | 0.9 |  |
| **-A*30** | 3 (4.5) | 15 (5.2) | 1.17 (0.33-4.16) | 0.81 | 1.11 (0.29-4.26) | 0.87 |  |
| **-A*31** | 1 (1.5) | 11 (3.8) | 2.61 (0.33-20.57) | 0.36 | 1.71 (0.2-14.58) | 0.62 |  |
| **-A*32** | 0 (0) | 7 (2.4) | 3718585.72 (0-Inf) | 0.99 | 5212070.98 (0-Inf) | 0.99 |  |
| **-A*33** | 6 (9) | 7 (2.4) | 0.25 (0.08-0.78) | 0.02 | 0.28 (0.09-0.91) | 0.03 |  |
| **-A*34** | 1 (1.5) | 2 (0.7) | 0.46 (0.04-5.15) | 0.53 | 0.32 (0.03-3.8) | 0.37 |  |
| **-A*36** | 2 (3) | 2 (0.7) | 0.23 (0.03-1.64) | 0.14 | 0.12 (0.02-0.9) | 0.04 |  |
| **-A*66** | 1 (1.5) | 5 (1.7) | 1.16 (0.13-10.11) | 0.89 | 1.12 (0.12-10.47) | 0.92 |  |
| **-A*68** | 9 (13.4) | 39 (13.5) | 1.01 (0.46-2.19) | 0.99 | 0.99 (0.44-2.24) | 0.99 |  |
| **-A*74** | 3 (4.5) | 7 (2.4) | 0.53 (0.13-2.1) | 0.37 | 0.49 (0.11-2.15) | 0.35 |  |
| **-A*80** | 1 (1.5) | 0 (0) | - | - | - | - |  |
|  |  |  |  |  |  |  |  |
| **B Locus n (%)** |  |  |  |  |  |  |  |
| **-B*07** | 3 (7.3) | 9 (4.2) | 0.56 (0.15-2.17) | 0.4 | 0.37 (0.09-1.54) | 0.17 |  |
| **-B*08** | 4 (9.8) | 12 (5.7) | 0.55 (0.17-1.81) | 0.33 | 0.47 (0.13-1.64) | 0.23 |  |
| **-B*13** | 0 (0) | 2 (0.9) | - | - | - | - |  |
| **-B*14** | 2 (4.9) | 7 (3.3) | 0.67 (0.13-3.33) | 0.62 | 0.76 (0.14-4.06) | 0.75 |  |
| **-B*15** | 7 (17.1) | 19 (9) | 0.48 (0.19-1.22) | 0.12 | 0.43 (0.16-1.15) | 0.09 |  |
| **-B*18** | 4 (9.8) | 10 (4.7) | 0.46 (0.14-1.54) | 0.21 | 0.49 (0.14-1.73) | 0.27 |  |
| **-B*27** | 0 (0) | 8 (3.8) | - | - | - | - |  |
| **-B*35** | 5 (12.2) | 18 (8.5) | 0.67 (0.23-1.91) | 0.45 | 0.82 (0.27-2.49) | 0.73 |  |
| **-B*37** | - | - | - | - | - | - |  |
| **-B*38** | 1 (2.4) | 8 (3.8) | 1.57 (0.19-12.89) | 0.68 | 1.37 (0.16-11.94) | 0.77 |  |
| **-B*39** | 1 (2.4) | 2 (0.9) | 0.38 (0.03-4.3) | 0.44 | 0.32 (0.02-4.41) | 0.4 |  |
| **-B*40** | 2 (4.9) | 10 (4.7) | 0.97 (0.2-4.58) | 0.96 | 1.12 (0.21-5.91) | 0.9 |  |
| **-B*41** | 0 (0) | 4 (1.9) | - | - | - | - |  |
| **-B*42** | 1 (2.4) | 7 (3.3) | 1.37 (0.16-11.41) | 0.77 | 0.98 (0.11-8.84) | 0.98 |  |
| **-B*44** | 3 (7.3) | 13 (6.1) | 0.83 (0.23-3.04) | 0.78 | 0.85 (0.22-3.25) | 0.81 |  |
| **-B*45** | 1 (2.4) | 5 (2.4) | 0.97 (0.11-8.49) | 0.98 | 1.03 (0.11-9.52) | 0.98 |  |
| **-B*47** | - | - | - | - | - | - |  |
| **-B*48** | 0 (0) | 5 (2.4) | - | - | - | - |  |
| **-B*49** | 0 (0) | 3 (1.4) | - | - | - | - |  |
| **-B*50** | 3 (7.3) | 3 (1.4) | 0.18 (0.04-0.93) | 0.04 | 0.21 (0.04-1.21) | 0.08 |  |
| **-B*51** | 0 (0) | 15 (7.1) | - | - | - | - |  |
| **-B*52** | 1 (2.4) | 4 (1.9) | 0.77 (0.08-7.06) | 0.82 | 0.66 (0.07-6.44) | 0.72 |  |
| **-B*53** | 0 (0) | 15 (7.1) | - | - | - | - |  |
| **-B*55** | - | - |  |  |  |  |  |
| **-B*56** | 0 (0) | 1 (0.5) | - | - | - | - |  |
| **-B*57** | 2 (4.9) | 11 (5.2) | 1.07 (0.23-5) | 0.93 | 1.23 (0.24-6.18) | 0.8 |  |
| **-B*58** | 1 (2.4) | 18 (8.5) | 3.71 (0.48-28.61) | 0.21 | 3.24 (0.41-25.72) | 0.27 |  |
| **-B*81** | 0 (0) | 1 (0.5) | - | - | - | - |  |
| **-B*82** | 0 (0) | 2 (0.9) | - | - | - | - |  |
|  |  |  |  |  |  |  |  |
| **C Locus n (%)** |  |  |  |  |  |  |  |
| **-C*01** | 1 (2.8) | 3 (1.4) | 0.51 (0.05-5.02) | 0.56 | 0.69 (0.06-7.5) | 0.76 |  |
| **-C*02** | 1 (2.8) | 16 (7.6) | 2.89 (0.37-22.47) | 0.31 | 3.13 (0.39-25.37) | 0.29 |  |
| **-C*03** | 5 (13.9) | 21 (10) | 0.69 (0.24-1.96) | 0.49 | 0.72 (0.24-2.18) | 0.57 |  |
| **-C*04** | 7 (19.4) | 34 (16.2) | 0.8 (0.32-1.98) | 0.63 | 0.77 (0.3-1.96) | 0.59 |  |
| **-C*05** | 3 (8.3) | 9 (4.3) | 0.49 (0.13-1.91) | 0.31 | 0.36 (0.09-1.5) | 0.16 |  |
| **-C*06** | 4 (11.1) | 20 (9.5) | 0.84 (0.27-2.63) | 0.77 | 0.96 (0.29-3.11) | 0.94 |  |
| **-C*07** | 7 (19.4) | 51 (24.3) | 1.33 (0.55-3.22) | 0.53 | 1.28 (0.52-3.18) | 0.59 |  |
| **-C*08** | 1 (2.8) | 11 (5.2) | 1.93 (0.24-15.45) | 0.53 | 1.92 (0.23-15.84) | 0.54 |  |
| **-C*12** | 3 (8.3) | 12 (5.7) | 0.67 (0.18-2.49) | 0.55 | 0.64 (0.16-2.54) | 0.52 |  |
| **-C*14** | 0 (0) | 7 (3.3) | - | - | - | - |  |
| **-C*15** | 0 (0) | 8 (3.8) | - | - | - | - |  |
| **-C*16** | 3 (8.3) | 14 (6.7) | 0.79 (0.21-2.88) | 0.72 | 0.73 (0.19-2.8) | 0.64 |  |
| **-C*17** | 1 (2.8) | 4 (1.9) | 0.68 (0.07-6.26) | 0.73 | 0.71 (0.07-6.98) | 0.77 |  |
| **-C*18** | - | - | - | - | - | - |  |
|  |  |  |  |  |  |  |  |
| **DRB1 Locus n (%)** | |  |  |  |  |  |  |
| **-DRB1*01** | 7 (20.6) | 21 (10.4) | 0.45 (0.17-1.15) | 0.1 | 0.49 (0.19-1.3) | 0.15 |  |
| **-DRB1*03** | 4 (11.8) | 21 (10.4) | 0.87 (0.28-2.71) | 0.81 | 0.68 (0.21-2.23) | 0.53 |  |
| **-DRB1*04** | 4 (11.8) | 20 (9.9) | 0.82 (0.26-2.58) | 0.74 | 0.85 (0.27-2.71) | 0.78 |  |
| **-DRB1*07** | 6 (17.6) | 21 (10.4) | 0.54 (0.2-1.46) | 0.22 | 0.55 (0.2-1.52) | 0.25 |  |
| **-DRB1*08** | 1 (2.9) | 10 (5) | 1.72 (0.21-13.87) | 0.61 | 1.79 (0.22-14.73) | 0.59 |  |
| **-DRB1*09** | 1 (2.9) | 7 (3.5) | 1.18 (0.14-9.94) | 0.88 | 1.13 (0.13-9.69) | 0.91 |  |
| **-DRB1*10** | 3 (8.8) | 7 (3.5) | 0.37 (0.09-1.51) | 0.17 | 0.32 (0.07-1.44) | 0.14 |  |
| **-DRB1*11** | 3 (8.8) | 32 (15.8) | 1.95 (0.56-6.74) | 0.29 | 1.94 (0.55-6.8) | 0.3 |  |
| **-DRB1*12** | 0 (0) | 3 (1.5) | - | - | - | - | - |
| **-DRB1*13** | 1 (2.9) | 27 (13.4) | 5.09 (0.67-38.74) | 0.12 | 5.71 (0.74-44.14) | 0.1 |  |
| **-DRB1*14** | 1 (2.9) | 6 (3) | 1.01 (0.12-8.66) | 0.99 | 0.99 (0.11-9.22) | 0.99 |  |
| **-DRB1*15** | 2 (5.9) | 19 (9.4) | 1.66 (0.37-7.48) | 0.51 | 1.49 (0.32-6.88) | 0.61 |  |
| **-DRB1*16** | 1 (2.9) | 8 (4) | 1.36 (0.16-11.24) | 0.77 | 1.61 (0.19-13.77) | 0.66 |  |
| HAM/TSP= Human T cell lymphotropic virus-associated myelopathy/tropical spastic paraparesis, OR= odds ratio 95% confidence interval, * *p* values adjusted by age, sex, ethnicity and time of clinical follow-up. | | | | | | |  |

| Supplemental Table S7. Frequency of HLA-A, B, C and DRB1 loci in HAM/TSP patients related to death outcome. | | | | | | | |
| --- | --- | --- | --- | --- | --- | --- | --- |
|  | **Death** | |  |  |  |  |  |
|  | **No (164)** | **Yes (24)** | **OR (95%CI)** | **P-value** | **OR-A (95%CI)*** | **P-value*** |  |
| **A Locus n (%)** |  |  |  |  |  |  |  |
| **-A*01** | 26 (7) | 0 (0) | - | - | - | - |  |
| **-A*02** | 64 (17.3) | 11 (26.2) | 1.7 (0.81-3.55) | 0.16 | 1.58 (0.74-3.38) | 0.23 |  |
| **-A*03** | 34 (9.2) | 3 (7.1) | 0.76 (0.22-2.59) | 0.66 | 0.88 (0.25-3.07) | 0.84 |  |
| **-A*11** | 28 (7.6) | 3 (7.1) | 0.94 (0.27-3.23) | 0.92 | 1.05 (0.29-3.81) | 0.94 |  |
| **-A*23** | 25 (6.8) | 2 (4.8) | 0.69 (0.16-3.02) | 0.62 | 0.52 (0.12-2.35) | 0.4 |  |
| **-A*24** | 38 (10.3) | 3 (7.1) | 0.67 (0.2-2.28) | 0.52 | 0.84 (0.24-2.93) | 0.78 |  |
| **-A*25** | 1 (0.3) | 0 (0) | - | - | - | - |  |
| **-A*26** | 15 (4.1) | 2 (4.8) | 1.18 (0.26-5.36) | 0.83 | 1.19 (0.25-5.7) | 0.82 |  |
| **-A*29** | 12 (3.2) | 1 (2.4) | 0.73 (0.09-5.74) | 0.76 | 0.62 (0.07-5.25) | 0.66 |  |
| **-A*30** | 21 (5.7) | 2 (4.8) | 0.83 (0.19-3.67) | 0.81 | 0.82 (0.18-3.73) | 0.8 |  |
| **-A*31** | 15 (4.1) | 1 (2.4) | 0.58 (0.07-4.48) | 0.6 | 0.81 (0.1-6.59) | 0.85 |  |
| **-A*32** | 7 (1.9) | 3 (7.1) | 3.99 (0.99-16.05) | 0.05 | 4.22 (0.97-18.36) | 0.06 |  |
| **-A*33** | 13 (3.5) | 1 (2.4) | 0.67 (0.09-5.25) | 0.7 | 0.55 (0.07-4.49) | 0.58 |  |
| **-A*34** | 3 (0.8) | 1 (2.4) | 2.98 (0.3-29.35) | 0.35 | 4.23 (0.42-42.88) | 0.22 |  |
| **-A*36** | 4 (1.1) | 0 (0) | - | - | - | - |  |
| **-A*66** | 7 (1.9) | 0 (0) | - | - | - | - |  |
| **-A*68** | 48 (13) | 7 (16.7) | 1.34 (0.56-3.19) | 0.51 | 1.21 (0.49-2.95) | 0.68 |  |
| **-A*74** | 8 (2.2) | 2 (4.8) | 2.26 (0.46-11.02) | 0.31 | 1.98 (0.39-10.07) | 0.41 |  |
| **-A*80** | 1 (0.3) | 0 (0) | - | - | - | - |  |
|  |  |  |  |  |  |  |  |
| **B Locus n (%)** |  |  |  |  |  |  |  |
| **-B*07** | 13 (5.2) | 0 (0) | - | - | - | - |  |
| **-B*08** | 15 (6) | 1 (3.2) | 0.53 (0.07-4.13) | 0.54 | 0.5 (0.06-4.27) | 0.53 |  |
| **-B*13** | 2 (0.8) | 0 (0) | - | - | - | - |  |
| **-B*14** | 10 (4) | 0 (0) | - | - | - | - |  |
| **-B*15** | 28 (11.1) | 3 (9.7) | 0.86 (0.24-3) | 0.81 | 0.97 (0.26-3.63) | 0.96 |  |
| **-B*18** | 16 (6.3) | 0 (0) | - | - | - | - |  |
| **-B*27** | 8 (3.2) | 0 (0) | - | - | - | - |  |
| **-B*35** | 24 (9.5) | 3 (9.7) | 1.02 (0.29-3.6) | 0.98 | 1.03 (0.27-3.87) | 0.96 |  |
| **-B*37** | 7 (2.8) | 3 (9.7) | 3.75 (0.92-15.33) | 0.07 | 7.12 (1.48-34.32) | 0.01 |  |
| **-B*38** | 3 (1.2) | 1 (3.2) | 2.77 (0.28-27.45) | 0.38 | 3.47 (0.33-36.41) | 0.3 |  |
| **-B*39** | 11 (4.4) | 2 (6.5) | 1.51 (0.32-7.16) | 0.6 | 2.29 (0.44-11.92) | 0.33 |  |
| **-B*40** | 4 (1.6) | 0 (0) | - | - | - | - |  |
| **-B*41** | 7 (2.8) | 1 (3.2) | 1.17 (0.14-9.81) | 0.89 | 2.37 (0.25-22.47) | 0.45 |  |
| **-B*42** | 16 (6.3) | 2 (6.5) | 1.02 (0.22-4.65) | 0.98 | 1 (0.21-4.8) | 1 |  |
| **-B*44** | 4 (1.6) | 2 (6.5) | 4.28 (0.75-24.37) | 0.1 | 3.75 (0.62-22.73) | 0.15 |  |
| **-B*45** | 8 (3.2) | 0 (0) | - | - | - | - |  |
| **-B*47** | 4 (1.6) | 1 (3.2) | 2.07 (0.22-19.1) | 0.52 | 1.01 (0.08-12.42) | 0.99 |  |
| **-B*48** | 7 (2.8) | 1 (3.2) | 1.17 (0.14-9.81) | 0.89 | 1.04 (0.11-9.53) | 0.97 |  |
| **-B*49** | 14 (5.6) | 4 (12.9) | 2.52 (0.77-8.2) | 0.13 | 2.1 (0.59-7.39) | 0.25 |  |
| **-B*50** | 4 (1.6) | 2 (6.5) | 4.28 (0.75-24.37) | 0.1 | 3.37 (0.54-21.17) | 0.19 |  |
| **-B*51** | 14 (5.6) | 1 (3.2) | 0.57 (0.07-4.46) | 0.59 | 0.56 (0.07-4.55) | 0.58 |  |
| **-B*52** | 0 (0) | 1 (3.2) | - | - | - | - |  |
| **-B*53** | 12 (4.8) | 1 (3.2) | 0.67 (0.08-5.31) | 0.7 | 0.82 (0.1-6.84) | 0.85 |  |
| **-B*55** | 19 (7.5) | 1 (3.2) | 0.41 (0.05-3.16) | 0.39 | 0.42 (0.05-3.41) | 0.42 |  |
| **-B*56** | 0 (0) | 1 (3.2) | - | - | - | - |  |
| **-B*57** | 2 (0.8) | 0 (0) | - | - | - | - |  |
| **-B*58** | 31 (6) | 4 (5.3) | 0.88 (0.3-2.56) | 0.81 | 0.93 (0.3-2.83) | 0.89 |  |
| **-B*81** | 7 (1.4) | 1 (1.3) | 0.98 (0.12-8.07) | 0.98 | 0.83 (0.09-8.11) | 0.87 |  |
| **-B*82** | 3 (0.6) | 1 (1.3) | 2.3 (0.24-22.42) | 0.47 | 1.69 (0.15-19.43) | 0.67 |  |
|  |  |  |  |  |  |  |  |
| **C Locus n (%)** |  |  |  |  |  |  |  |
| **-C*01** | 4 (1.6) | 0 (0) | - | - | - | - |  |
| **-C*02** | 20 (8.2) | 2 (6.2) | 0.75 (0.17-3.35) | 0.7 | 0.63 (0.13-3.02) | 0.56 |  |
| **-C*03** | 25 (10.2) | 4 (12.5) | 1.25 (0.41-3.86) | 0.7 | 1.07 (0.33-3.48) | 0.91 |  |
| **-C*04** | 40 (16.4) | 6 (18.8) | 1.18 (0.46-3.04) | 0.74 | 1.35 (0.5-3.69) | 0.55 |  |
| **-C*05** | 14 (5.7) | 0 (0) | - | - | - | - |  |
| **-C*06** | 23 (9.4) | 3 (9.4) | 0.99 (0.28-3.52) | 0.99 | 0.85 (0.23-3.16) | 0.81 |  |
| **-C*07** | 59 (24.2) | 4 (12.5) | 0.45 (0.15-1.33) | 0.15 | 0.45 (0.14-1.39) | 0.17 |  |
| **-C*08** | 14 (5.7) | 0 (0) | - | - | - | - |  |
| **-C*12** | 11 (4.5) | 5 (15.6) | 3.92 (1.27-12.14) | 0.02 | 6.25 (1.71-22.8) | 0.01 |  |
| **-C*14** | 4 (1.6) | 4 (12.5) | 8.57 (2.03-36.18) | <0.01 | 8.85 (1.68-46.67) | 0.01 |  |
| **-C*15** | 8 (3.3) | 1 (3.1) | 0.95 (0.12-7.87) | 0.96 | 1.84 (0.2-17.25) | 0.59 |  |
| **-C*16** | 18 (7.4) | 2 (6.2) | 0.84 (0.18-3.79) | 0.82 | 0.73 (0.15-3.52) | 0.69 |  |
| **-C*17** | 4 (1.6) | 1 (3.1) | 1.94 (0.21-17.87) | 0.56 | 1.53 (0.15-15.49) | 0.72 |  |
|  |  |  |  |  |  |  |  |
| **DRB1 Locus n (%)** | |  |  |  |  |  |  |
| **-DRB1*01** | 28 (11.2) | 2 (11.1) | 0.99 (0.22-4.54) | 0.99 | 0.54 (0.1-2.84) | 0.47 |  |
| **-DRB1*03** | 29 (11.6) | 0 (0) | - | - | - | - |  |
| **-DRB1*04** | 28 (11.2) | 2 (11.1) | 0.99 (0.22-4.54) | 0.99 | 0.77 (0.15-4.01) | 0.76 |  |
| **-DRB1*07** | 28 (11.2) | 2 (11.1) | 0.99 (0.22-4.54) | 0.99 | 1.49 (0.3-7.41) | 0.62 |  |
| **-DRB1*08** | 9 (3.6) | 3 (16.7) | 5.36 (1.31-21.87) | 0.02 | 9.1 (1.72-48.04) | 0.01 |  |
| **-DRB1*09** | 8 (3.2) | 1 (5.6) | 1.78 (0.21-15.07) | 0.6 | 1.2 (0.12-11.89) | 0.87 |  |
| **-DRB1*10** | 11 (4.4) | 0 (0) | - | - | - | - |  |
| **-DRB1*11** | 39 (15.6) | 1 (5.6) | 0.32 (0.04-2.46) | 0.27 | 0.29 (0.03-2.36) | 0.25 |  |
| **-DRB1*12** | 4 (1.6) | 1 (5.6) | 3.62 (0.38-34.18) | 0.26 | 3.92 (0.35-44.55) | 0.27 |  |
| **-DRB1*13** | 30 (12) | 1 (5.6) | 0.43 (0.06-3.36) | 0.42 | 0.43 (0.05-3.54) | 0.43 |  |
| **-DRB1*14** | 7 (2.8) | 1 (5.6) | 2.04 (0.24-17.57) | 0.52 | 1.64 (0.17-15.81) | 0.67 |  |
| **-DRB1*15** | 20 (8) | 2 (11.1) | 1.44 (0.31-6.7) | 0.64 | 2.98 (0.56-15.94) | 0.2 |  |
| **-DRB1*16** | 9 (3.6) | 2 (11.1) | 3.35 (0.67-16.81) | 0.14 | 2.81 (0.48-16.53) | 0.25 |  |
| HAM/TSP= Human T cell lymphotropic virus-associated myelopathy/tropical spastic paraparesis, OR= odds ratio 95% confidence interval, * *p* values adjusted by age, sex, ethnicity and time of clinical follow-up. | | | | | | |  |
