# Supplementary material for: Putative role of HLA polymorphism among a Brazilian HTLV-1-associated myelopathy/tropical spastic paraparesis (HAM/TSP) population
Source: Sci Rep. 2023 May 11;13:7659. doi: 10.1038/s41598-023-34757-w (PMC10173239; doi:10.1038/s41598-023-34757-w)
Supplement: Supplementary file 2 — Supplementary Information 2. [file 41598_2023_34757_MOESM2_ESM.docx]

| **Supplemental Table S2 . Hardy-Weinberg equilibrium estimates in Asymptomatic HTLV-1-infected patients** |
| --- |
| Locus Obs.Het. Exp.Het. P-value s.d. Steps done |
| A 0.87578 0.90929 0.08931 0.00019 1001000 |
| B 0.89032 0.94018 0.00000 0.00000 1001000 |
| C 0.80132 0.87734 0.09065 0.00028 1001000 |
| DRB1 0.89189 0.89237 0.08160 0.00020 1001000 |
| DQA1 0.77174 0.73390 0.75834 0.00040 1001000 |
| DQB1 0.80000 0.76397 0.98625 0.00011 1001000 |
| Obs.Het.=Observed heterozygosity, Exp.Het=expectected heterozygosity, ±SD=standard deviation. The data represents the frequency of each HLA locus in the AC group.  **Supplemental Table S3. Hardy-Weinberg equilibrium estimates in HAM/TSP patients**   \| Locus Obs.Het. Exp.Het. P-value s.d. Steps done \| \| --- \| \| A 0.88679 0.90661 0.36151 0.00030 1001000 \| \| B 0.89933 0.94753 0.00088 0.00003 1001000 \| \| C 0.82119 0.88152 0.04560 0.00019 1001000 \| \| DRB1 0.90511 0.90329 0.70725 0.00037 1001000 \| \| DQA1 0.82692 0.71322 0.19412 0.00039 1001000 \| \| DQB1 0.84536 0.76898 0.25026 0.00041 1001000 \| |

Obs.Het.=Observed heterozygosity, Exp.Het=expectected heterozygosity, ±SD=standard deviation. The data represents the frequency of each HLA locus in the HAM/TSP group.
